# Association between Blood Lead and Walking Speed in the National Health and Nutrition Examination Survey (NHANES 1999–2002)

**DOI:** 10.1289/ehp.1205918

**Published:** 2013-04-19

**Authors:** John S. Ji, Alexis Elbaz, Marc G. Weisskopf

**Affiliations:** 1 Department of Environmental Health, Harvard School of Public Health, Boston, Massachusetts, USA; 2 INSERM, Centre for Research in Epidemiology and Population Health, U1018, Social and Occupational Determinants of Health, F-94807, Villejuif, France; 3 Université de Versailles St-Quentin, UMRS 1018, F-78035, Versailles, France; 4 Department of Epidemiology, Harvard School of Public Health, Boston, Massachusetts, USA

**Keywords:** environmental epidemiology, gait speed, lead, NHANES, toxicant, walking speed

## Abstract

Background: Walking speed is a simple and reliable measure of motor function that is negatively associated with adverse health events in older people, including falls, disability, hospital admissions, and mortality. Lead has adverse affects on human health, particularly on the vascular and neurological systems.

Objective: We explored the hypothesis that lead is associated with slower walking speed.

Methods: We used U.S. National Health and Nutrition Examination Survey (NHANES) cross-sectional data from 1999–2002. The time to walk 20 ft (walking speed) was measured among 1,795 men and 1,798 women ≥ 50 years of age. The association between walking speed and quintiles of blood lead concentration was estimated separately in men and women using linear regression models adjusted for age, education, ethnicity, alcohol use, smoking status, height, and waist circumference.

Results: Mean blood lead concentrations and walking speeds were 2.17 μg/dL and 3.31 ft/sec in women, and 3.18 μg/dL and 3.47 ft/sec in men, respectively. Among women, walking speed decreased with increasing quintiles of blood lead, resulting in an estimated mean value that was 0.11 ft/sec slower (95% CI: –0.19, –0.04; *p*-trend = 0.005) for women with blood lead concentrations in the highest versus lowest quintile. In contrast, lead was not associated with walking speed in men.

Conclusion: Blood lead concentration was associated with decreased walking speed in women, but not in men. Our results contribute to the growing evidence that lead exposure, even at low levels, is detrimental to public health.

Decline of motor performance is an important feature of the aging process. Walking speed is a reliable measure of functional mobility and motor performance in elderly people and has been associated with adverse health effects, including falls, late-life disability, hospital admissions, and mortality (Dumurgier et al. 2009; Kuo et al. 2006b). Cognitive function, particularly executive function, appears intimately connected with motor function (Hausdorff et al. 2005; Rosano et al. 2005; Sheridan et al. 2003). There is increasing evidence that vascular risk factors (hypertension) (Dumurgier et al. 2010), metabolic conditions (diabetes) (Ko et al. 2011), and markers of subclinical atherosclerosis (Brunner et al. 2011; Elbaz et al. 2005) are negatively associated with motor performance, which is thought to be a result of their effects on the nervous system, such as increases in white matter lesions (Soumare et al. 2009a).

Lead is detrimental in many aspects of human health. Even at general environmental exposure levels, lead exposure is associated with lower IQ during neurodevelopment (Lanphear et al. 2005) and impaired cognitive function among adults (Bandeen-Roche et al. 2009; Shih et al. 2006, 2007; Weisskopf et al. 2007; Weuve et al. 2009). In addition, there is some evidence of an association between Parkinson’s disease and lead exposure (Weisskopf et al. 2010). Neurological consequences of lead exposure may be attributable to direct neurotoxic effects on the vascular system, including hypertension and cardiovascular disease, or through effects on diabetes and poor renal health (Menke et al. 2006; Navas-Acien et al. 2007; Schober et al. 2006; Weisskopf et al. 2009).

Given the neurological and vascular toxicities of lead (Baker et al. 1979; Menke et al. 2006; Navas-Acien et al. 2007; Schober et al. 2006; Shih et al. 2007; Tsaih et al. 2004; Weisskopf et al. 2009, 2010) and the association between occupational lead exposure and neuromuscular function (Baker et al. 1979; Bleecker et al. 2007; Weisskopf et al. 2004), we wanted to explore whether lead exposure at general environmental exposure levels could adversely affect walking speed. We therefore studied the cross-sectional relation between walking speed and blood lead concentration among participants in the National Health and Nutrition Examination Survey (NHANES), a population of randomly selected civilian, noninstitutionalized residents across the United States.

## Methods

*Study population*. Beginning in the 1960s, the National Center for Health Statistics conducted a series of cross-sectional health and nutrition surveys known as the National Health and Nutrition Examination Survey (NHANES). The NHANES study protocol is described in detail elsewhere (Centers for Disease Control and Prevention 2012). In brief, NHANES uses a complex, multistage probability sampling design, with oversampling of adolescents 12–19 years of age, adults ≥ 60 years of age, low-income persons, Mexican Americans, and non-Hispanic blacks. All participants completed household surveys conducted by trained study staff, which include questions about demographics and health history. The study protocol also includes standardized medical examinations, collection of a blood sample, and other in-person testing at mobile examination centers. Informed consent was obtained from all NHANES participants.

For the present study we used publicly available NHANES data from the 1999–2000 and 2001–2002 surveys, in which time to walk 20 ft (at usual walking pace) was measured in participants ≥ 50 years of age. We excluded participants (*n* = 230) who had had a myocardial infarction in the preceding 6 weeks; chest or abdominal surgery within the preceding 3 weeks; a history of brain aneurysm or stroke, knee surgery or knee replacement, or severe back pain; used an assistive device during walking speed measurement; or had Parkinson’s disease or reported taking Parkinson’s medication. We also excluded four people with improbably high walking speed (> 6 ft/sec). Our final sample for blood lead and walking speed consisted of 1,798 women and 1,795 men. Our study was deemed exempt from full review by the Institutional Review Board of the Harvard School of Public Health.

*Blood lead assessment*. We determined blood lead concentration using atomic absorption spectroscopy on a PerkinElmer Model SIMAA 6000 while employing Zeeman background correction (PerkinElmer, Norwalk, CT, USA). Whole blood specimens were processed, stored, and shipped to the Division of Laboratory Sciences, National Center for Environmental Health, Centers for Disease Control and Prevention (Atlanta, GA, USA), for analysis. Vials were stored at –20°C until they were shipped. The limit of detection (LOD) was 0.3 μg/dL (Miller et al. 1987; Parsons and Slavin 1993). NHANES quality assurance and quality control protocols meet the 1988 Clinical Laboratory Improvement Act mandates (Centers for Disease Control and Prevention 2009).

*Outcome assessment*. Walking speed was derived from the measured walk component of the muscle strength module completed as part of a medical examination at an NHANES mobile examination center. Measurements were performed by certified health technicians who received intensive training on NHANES examination protocols (Bohannon 2008). Subjects were asked to walk at their usual pace. They began with their toes behind a starting line. After subjects were told to start, timing with a hand-held stopwatch began when their first foot touched the floor beyond the start line. A 20-ft stop time was obtained when the first foot touched the floor beyond the 20-ft finish line. Walking speed has been shown to have excellent test–retest reliability in several past studies (Bohannon 1997; Graham et al. 2008; Ostir et al. 2002).

*Covariates*. Demographic variables included age, sex, education (less than 9th grade, 9th–11th grade, high school graduate, some college or associate degree, college graduate and above), and ethnicity (Mexican American, other Hispanic, non-Hispanic white, non-Hispanic black, and other). To avoid identifiability, NHANES indicates age as 85 years for all individuals > 85 years of age (4.3% of our study population). Anthropometric covariates were standing height and waist circumference (categorized into tertiles), which are strongly associated with walking speed (Bohannon 2008).

Lifestyle variables were smoking (never, past, current), alcohol consumption (none, past, current light, current heavy), and physical activity. Participants who smoked ≥ 100 cigarettes or a pipe or cigar ≥ 20 times in their lifetime were classified as current smokers if they reported currently smoking “some days” or “every day,” and were classified as former smokers if they currently smoked “not at all”; otherwise they were classified as never smokers. Current drinkers were classified as heavy drinkers if they consumed more than one drink a day (defined as a 10-oz beer, a 4-oz glass of wine, or 1 oz of liquor) on average, and as light drinkers if they consumed one drink a day or less on average. Participants who consumed 12 drinks in their lifetime, but did not consume alcohol in the past year, were considered past drinkers. Physical activity was classified according to tertiles of metabolic equivalents task scores based on the intensity and frequency of a number of physical activities reported during in the preceding 30 days (Ainsworth et al. 1993).

Some clinical comorbidities have been associated with slower walking speed (Atkinson et al. 2007; Dumurgier et al. 2010) including diabetes, arthritis, and heart conditions (angina, myocardial infarction, coronary heart disease, congestive heart failure). These variables were considered separately because they could also be mediators of effects of lead exposure on walking speed. Participants were classified as not having a condition if they answered “don’t know” when asked about their history of the condition (< 0.5% for any of the conditions). In addition, we adjusted for hypertension, which was classified based on self-reported current use of medication for high blood pressure, or mean systolic blood pressure ≥ 140 mmHg or mean diastolic blood pressure ≥ 90 mmHg based on up to four blood pressure measurements during the NHANES examination. In addition, we adjusted for serum homocysteine and C-reactive protein concentrations as biomarkers of tissue damage and inflammation. These inflammatory markers have been associated with slower walking speed (Albert et al. 2002; Kuo et al. 2006a, 2007b), but also could be mediators of the effects of lead.

Finally, we considered scores on the Digit Symbol Substitution Test (DSST) (Wechsler 1997) as a measure of cognitive function, which were available for participants ≥ 60 years of age. Specifically, the DSST is a test of visuospatial and psychomotor speed that is frequently used as a measure of frontal lobe executive function, and has been negatively associated with walking speed (Kuo et al. 2007a).

*Statistical analysis*. We used SAS, version 9.2 (SAS Institute Inc., Cary, NC, USA) for all analyses. We combined NHANES cycles using NHANES cluster design variables (SDMVSTRA, SDMVPSU) and medical examination weights (WTME-C4YR) for 1999–2002.

We used PROC SURVEYREG to perform separate adjusted linear regressions for men and women with walking speed as the dependent variable. Blood lead concentration was divided into quintiles based on its distribution in both women and men. Analyses were stratified by sex because blood lead levels are higher in men than in women (Vahter et al. 2007) and men walk faster than women (Cooper et al. 2011). We explored possible nonlinear associations using piecewise regression with a 4-knot cubic spline for lead (STATA, version 11.0; StataCorp., College Station, TX, USA), but there was no evidence of departure from linearity in women (*p* = 0.27) or men (*p* = 0.13). To reduce the influence of extreme exposure levels on linear trends, we tested for trend by modeling a continuous blood lead variable coded as the median value of each blood lead quintile.

Our first model (model 1) was adjusted for the demographic (age, sex, education, and ethnicity) and anthropometric variables (height and waist circumference) only, because these are very strongly associated with walking speed (Bohannon 2008). In addition, we conducted a model that also included the lifestyle variables (smoking, alcohol consumption, and physical activity) (model 2); a model that included the demographic and anthropomorphic variables plus medical comorbidities (diabetes, arthritis, heart conditions, and hypertension) and the serum biomarkers of inflammation (homocysteine and C-reactive protein) (model 3); and a fully adjusted model that included all of the covariates above (model 4). In addition, we conducted sensitivity analyses adjusting for DSST score as a measure of cognitive function, and analyses that excluded participants with blood lead concentrations ≥ 10 μg/dL or ≥ 5 μg/dL.

## Results

The relation between mean blood lead and walking speed and participants’ characteristics is shown separately for women and men in Table 1. Study participants had a median age of 61.2 years (women: 62.1; men: 60.0), with a range of 50 to at least 85 years. Blood lead was lower in women (mean ± SD, 2.17 ± 0.06 μg/dL; median, 1.72 μg/dL) than in men (mean, 3.18 ± 0.12 μg/dL; median, 2.41 μg/dL). Blood lead levels tended to be higher in women and men who were older, non-Hispanic black, or smokers, and in those with lower education, higher alcohol consumption, and higher serum homocysteine concentrations. The mean walking speed for women was 3.31 ± 0.03 ft/sec, and for men was 3.47 ± 0.03 ft/sec. Walking speed tended to be slower in women and men who were older or shorter, or were smokers; in those with hypertension, diabetes, arthritis, or a heart condition; and in those with a larger waist circumference, lower alcohol consumption, lower education, higher serum homocysteine, or higher C-reactive protein.

**Table 1:** Blood lead concentration and walking speed (mean ± SD) for women and men according to demographic, anthropomorphic, lifestyle, and medical history.

Characteristics	Women			Men		
	Participants [*n* (%)]	Blood lead (μg/dL)	Walking speed (ft/sec)	Participants [*n* (%)]	Blood lead (μg/dL)	Walking speed (ft/sec)
Overall	1,798 (100)	2.17 ± 0.04	3.31 ± 0.03	1795	3.18 ± 0.08	3.47 ± 0.03
Age (years)
50–54	308 (17.1)	1.84 ± 0.05	3.69 ± 0.06	303 (16.9)	2.79 ± 0.10	3.77 ± 0.05
55–59	213 (11.8)	1.99 ± 0.08	3.59 ± 0.06	217 (12.1)	2.74 ± 0.14	3.64 ± 0.05
60–64	354 (19.7)	2.09 ± 0.12	3.33 ± 0.05	328 (18.3)	3.28 ± 0.37	3.53 ± 0.05
65–69	271 (15.1)	2.06 ± 0.12	3.26 ± 0.06	271 (15.1)	3.02 ± 0.12	3.45 ± 0.07
70–74	246 (13.7)	2.25 ± 0.10	3.05 ± 0.07	253 (14.1)	3.34 ± 0.26	3.25 ± 0.06
75–79	162 (9.0)	2.23 ± 0.12	2.94 ± 0.08	183 (10.2)	3.02 ± 0.14	3.04 ± 0.04
≥ 80	244 (13.6)	2.50 ± 0.10	2.57 ± 0.05	240 (13.4)	3.42 ± 0.10	2.74 ± 0.05
Education
Less than 9th grade	355 (19.8)	2.47 ± 0.15	2.67 ± 0.05	385 (21.5)	3.53 ± 0.17	3.00 ± 0.05
9th–11th grade	315 (17.6)	2.14 ± 0.12	3.06 ± 0.05	291 (16.2)	3.60 ± 0.14	3.21 ± 0.06
High school graduate	453 (25.3)	2.14 ± 0.08	3.25 ± 0.04	364 (20.3)	3.02 ± 0.10	3.38 ± 0.03
Some college or associate’s degree	424 (23.6)	1.88 ± 0.05	3.45 ± 0.04	358 (20.0)	3.03 ± 0.26	3.55 ± 0.05
College graduate and above	246 (13.7)	2.01 ± 0.08	3.78 ± 0.05	393 (21.9)	2.53 ± 0.14	3.78 ± 0.04
Ethnicity
Mexican American	362 (21.1)	2.13 ± 0.17	3.13 ± 0.07	347 (19.3)	3.10 ± 0.15	3.22 ± 0.05
Other Hispanic	84 (4.7)	2.17 ± 0.15	2.83 ± 0.08	73 (4.1)	3.86 ± 1.37	3.29 ± 0.10
Whites (non–Hispanic)	1,010 (56.2)	2.04 ± 0.05	3.38 ± 0.04	1,066 (59.4)	2.89 ± 0.07	3.54 ± 0.03
Black (non–Hispanic)	290 (16.1)	2.42 ± 0.10	2.98 ± 0.04	272 (15.2)	4.00 ± 0.25	3.11 ± 0.04
Other	52 (2.9)	2.11 ± 0.24	3.24 ± 0.16	37 (2.1)	2.51 ± 0.21	3.28 ± 0.12
Smoking
Never smoker	1,063 (59.3)	1.91 ± 0.05	3.28 ± 0.04	461 (25.7)	2.71 ± 0.25	3.55 ± 0.04
Past smoker	485 (27.0)	2.17 ± 0.07	3.36 ± 0.05	924 (51.6)	2.90 ± 0.07	3.48 ± 0.03
Current smoker	244 (13.6)	2.51 ± 0.09	3.31 ± 0.04	372 (20.8)	3.57 ± 0.11	3.42 ± 0.04
Alcohol use
Never drinker	465 (26.3)	1.90 ± 0.05	3.03 ± 0.06	122 (6.9)	2.13 ± 0.17	3.34 ± 0.07
Past drinker	464 (26.2)	2.08 ± 0.08	3.22 ± 0.05	233 (13.1)	2.60 ± 0.11	3.39 ± 0.07
Current drinker light	605 (34.2)	2.03 ± 0.07	3.53 ± 0.04	790 (44.6)	2.98 ± 0.16	3.56 ± 0.03
Current drinker heavy	81 (4.6)	2.81 ± 0.18	3.66 ± 0.07	278 (15.7)	3.70 ± 0.12	3.62 ± 0.05
Height (cm)^*a*^
Tertile 1	604 (34.0)	2.22 ± 0.08	2.99 ± 0.04	590 (33.4)	3.25 ± 0.25	3.26 ± 0.04
Tertile 2	583 (32.8)	2.09 ± 0.07	3.32 ± 0.07	587 (33.3)	3.04 ± 0.13	3.45 ± 0.05
Tertile 3	589 (33.2)	1.96 ± 0.05	3.52 ± 0.04	588 (33.3)	2.85 ± 0.07	3.66 ± 0.04
Waist circumference (cm)^*b*^
Tertile 1	595 (33.5)	2.17 ± 0.08	3.53 ± 0.05	591 (33.4)	3.44 ± 0.23	3.53 ± 0.04
Tertile 2	595 (33.5)	2.06 ± 0.08	3.31 ± 0.05	590 (33.4)	2.88 ± 0.10	3.54 ± 0.04
Tertile 3	588 (33.1)	2.01 ± 0.06	3.09 ± 0.04	588 (33.2)	2.75 ± 0.08	3.39 ± 0.05
Homocysteine (μmol/L)^*c*^
Tertile 1	599 (33.4)	1.78 ± 0.04	3.53 ± 0.04	598 (33.4)	2.61 ± 0.10	3.66 ± 0.04
Tertile 2	596 (33.3)	2.02 ± 0.06	3.32 ± 0.05	594 (33.2)	3.00 ± 0.18	3.48 ± 0.05
Tertile 3	596 (33.3)	2.47 ± 0.08	3.04 ± 0.06	596 (33.3)	3.52 ± 0.12	3.25 ± 0.04
C–reactive protein (mg/dL)^*d*^
Tertile 1	612 (34.7)	2.19 ± 0.07	3.44 ± 0.05	616 (34.7)	2.76 ± 0.08	3.63 ± 0.03
Tertile 2	573 (32.5)	2.09 ± 0.06	3.33 ± 0.04	590 (33.3)	3.20 ± 0.22	3.47 ± 0.04
Tertile 3	580 (32.9)	1.94 ± 0.07	3.14 ± 0.04	567 (32.0)	3.12 ± 0.09	3.30 ± 0.04
Metabolic equivalents task score^*e*^
Tertile 1	752 (41.8)	2.42 ± 0.06	2.85 ± 0.05	537 (29.9)	3.47 ± 0.17	2.97 ± 0.06
Tertile 2	504 (28.0)	2.03 ± 0.06	3.31 ± 0.04	609 (33.9)	3.25 ± 0.15	3.37 ± 0.04
Tertile 3	542 (30.1)	2.09 ± 0.07	3.41 ± 0.03	649 (36.2)	3.10 ± 0.08	3.43 ± 0.03
Hypertension
Yes	819 (45.6)	2.15 ± 0.07	3.13 ± 0.05	629 (35.0)	3.23 ± 0.12	3.35 ± 0.05
No	979 (54.4)	2.03 ± 0.04	3.43 ± 0.03	1,166 (65.0)	2.92 ± 0.11	3.53 ± 0.03
Diabetes
Yes	256 (14.2)	2.05 ± 0.10	2.91 ± 0.06	281 (15.7)	2.39 ± 0.13	3.17 ± 0.06
No	1,542 (85.8)	2.08 ± 0.04	3.36 ± 0.03	1,514 (84.3)	3.10 ± 0.09	3.52 ± 0.03
Arthritis
Yes	853 (47.4)	2.09 ± 0.06	3.09 ± 0.04	621 (34.6)	2.98 ± 0.10	3.37 ± 0.04
No	945 (52.6)	2.07 ± 0.05	3.50 ± 0.03	1,174 (65.4)	3.03 ± 0.11	3.54 ± 0.05
Heart condition
Yes	227 (12.6)	2.31 ± 0.14	2.93 ± 0.08	344 (19.2)	2.87 ± 0.09	3.22 ± 0.04
No	1,571 (87.4)	2.05 ± 0.04	3.36 ± 0.03	1,451 (80.8)	3.04 ± 0.10	3.54 ± 0.03
^***a***^Mean values ± SD in women and men, respectively: 160.7 ± 0.20 and 174.9 ± 0.15 cm. Tertile ranges (cm) in women and men, respectively: tertile 1, 25.6–156.4 and 136.7–156.4; tertile 2, 156.5–162.5 and 170.1–176.4; tertile 3, 162.6–183.9 and 176.5–195.6.^***b***^Mean values ± SD in women and men, respectively: 96.0 ± 0.55 and 103.4 ± 0.40 cm. Tertile ranges (cm) in women and men, respectively: tertile 1, 58.5–89.3 and 66.7–97.0; tertile 2, 89.4–101.8 and 97.1–107.1; tertile 3, 101.9–147.5 and 107.2–157.0.^***c***^Mean values ± SD in women and men, respectively: 9.07 ± 0.09 and 10.29 ± 0.11 μmol/L. Tertile ranges (μmol/L) in women and men, respectively: tertile 1, 1.65–7.33 and 2.97–8.61; tertile 2, 7.34–9.48 and 8.62–10.99; tertile 3, 9.49–156.3 and 11.00–83.64.^***d***^Mean values ± SD in women and men, respectively: 0.56 ± 0.02 and 0.42 ± 0.03 mg/dL. Tertile ranges (mg/dL) in women and men, respectively: tertile 1, 0.01–0.22 and 0.01–0.14; tertile 2, 0.22–0.54 and 0.15–0.36; tertile 3 0.55–29.6 and 0.37–16.00.^***e***^Mean values ± SD in women and men, respectively: 82.3 ± 75.9 and 90.2 ± 76.3 units. Tertile ranges (units) in women and men, respectively: tertile 1, 0–45 and 0–45; tertile 2, 16.5–82.5 and 46.0–82.5; tertile 3, 83.5–1398.0 and 83.0–1398.0.						

Among women, walking speed decreased with increasing quintiles of blood lead, and the highest quintile of lead was significantly different from the lowest based on all four models (Table 2). For example, in the fully adjusted model women in the highest quintile had a mean walking speed that was 0.114 ft/sec slower (95% CI: –0.191, –0.038) than women in the lowest quintile (trend *p*-value = 0.005). In this model, the reduction in walking speed associated with 5 more years of age was –0.127 ft/sec; 95% CI: –0.151, –0.104). We found similar results when restricting the analyses to women with DSST scores (*n* = 1,145) and adjusting for DSST score (data not shown). Results were essentially unchanged when we excluded women with blood lead concentrations ≥ 10 μg/dL (*n* = 7, 0.4%) or ≥ 5 μg/dL (*n* = 75, 4.2%) (data not shown).

**Table 2:** Blood lead concentration in relation to walking speed.

Quintile range (μg/dL)	Total [*n* (%)]	Model 1		Model 2		Model 3		Model 4	
		Mean change in walking speed (ft/sec) (95% CI)	*p*-Value	Mean change in walking speed (ft/sec) (95% CI)	*p*-Value	Mean change in walking speed (ft/sec) (95% CI)	*p*-Value	Mean change in walking speed (ft/sec) (95% CI)	*p*-Value
Women
0.2 to ≤ 1.2	413 (23.0)	0 (reference)	—	0 (reference)	—	0 (reference)	—	0 (reference)	—
1.3 to ≤ 1.6	348 (19.4)	–0.024 (–0.115, 0.066)	0.59	–0.021 (–0.113, 0.072)	0.65	–0.026 (–0.116, 0.063)	0.55	–0.024 (–0.112, 0.064)	0.58
1.7 to ≤ 2.1	341 (19.0)	–0.022 (–0.123, 0.080)	0.67	–0.018 (–0.108, 0.071)	0.68	–0.028 (–0.132, 0.077)	0.59	–0.027 (–0.118, 0.063)	0.54
2.2 to ≤ 2.9	356 (19.8)	–0.067 (–0.158, 0.024)	0.14	–0.097 (–0.184, 0.009)	0.03	–0.075 (–0.165, 0.015)	0.10	–0.104 (–0.187, –0.021)	0.02
3.0 to ≤ 53.0	340 (18.9)	–0.112 (–0.795, –0.029)	0.01	–0.117 (–0.191, 0.043)	0.01	–0.101 (–0.187, –0.015)	0.02	–0.114 (–0.191, –0.038)	0.01
Trend	1,798	—	0.006	—	0.002	—	0.015	—	0.005
Men
0.2 to ≤ 1.7	399 (22.2)	0 (reference)	—	—	—	0 (reference)	—	0 (reference)	—
1.8 to ≤ 2.3	364 (20.3)	0.056 (–0.043, 0.156)	0.25	0.061 (–0.047, 0.172)	0.27	0.036 (–0.062, 0.135)	0.46	0.057 (–0.050, 0.165)	0.29
2.4 to ≤ 3.0	342 (19.1)	0.054 (–0.039, 0.146)	0.24	0.020 (–0.075, 0.116)	0.67	0.042 (–0.051, 0.134)	0.36	0.017 (–0.073, 0.107)	0.71
3.1 to ≤ 4.3	354 (19.7)	0.053 (–0.035, 0.142)	0.23	0.065 (–0.032, 0.162)	0.18	0.057 (–0.036, 0.150)	0.22	0.082 (–0.012, 0.175)	0.08
4.4 to ≤ 54.0	336 (18.7)	–0.077 (–0.177, 0.024)	0.13	–0.047 (–0.162, 0.073)	0.43	–0.066 (–0.173, 0.041)	0.22	–0.029 (–0.154, 0.097)	0.64
Trend	1,795	—	0.03	—	0.34	—	0.10	—	0.60
Model 1: adjusted for demographic (age, education, ethnicity) and anthropometric variables (height and waist circumference). Model 2: model 1 covariates plus lifestyle variables (alcohol, smoking, physical activity). Model 3: model 1 covariates plus medical comorbidities and biomarkers (arthritis, diabetes, heart condition, hypertension, homocysteine, and C-reactive protein). Model 4: fully adjusted model.									

For men, the model adjusted for demographic and anthropometric variables only (model 1) indicated a statistically significant linear trend (*p* = 0.03) toward decreasing walking speed with increasing blood lead (Table 2). However, this was driven by a negative association for the highest versus lowest quintile of blood lead, because there were nonsignificant increases in mean walking speeds for men with blood lead concentrations in the 2nd–4th quintiles of lead compared with the 1st. The negative trend was no longer significant when adjusted for lifestyle variables or comorbidities (e.g., model 4, *p*-trend = 0.60). In analyses among men with DSST scores (*n* = 1,115), further adjustment for DSST scores did not meaningfully change the lead results, although lower DSST scores were strongly associated with slower walking speed (data not shown). Results were essentially unchanged when we excluded men with blood lead concentrations ≥ 10 μg/dL (*n* = 40, 2.2%) or ≥ 5 μg/dL (*n* = 263, 14.1%) (data not shown).

## Discussion

We found that walking speed decreased with increasing blood lead concentrations among women, but not among men. This association persisted with adjustment for age, education, ethnicity, smoking, alcohol use, physical activity, height, waist circumference, serum homocysteine, serum C-reactive protein, and several medical comorbidities. The estimated reduction in mean walking speed associated with the highest quintile compared to the lowest quintile of blood lead among women (–0.114 ft/sec; 95% CI: –0.191, –0.038) was approximately equivalent to the estimated reduction in walking speed associated with 5 more years of age among women in our NHANES data (–0.127 ft/sec; 95% CI: –0.151, –0.104). Based on a recent meta-analysis of the relation between walking speed and mortality in populations with mean ages ranging from 72 to 79 years and mean walking speeds of 1.84–3.90 ft/sec (Studenski et al. 2011), the 0.114-ft/sec reduction in walking speed estimated for women in the highest quintile of lead would be associated with an approximate 4% increase in mortality, suggesting a clinically relevant difference. Our NHANES population had an average age of 61 years and an average walking speed of 3.39 ft/sec, consistent with the studies included in the meta-analysis.

There is evidence that health effects of metals can differ between men and women (Vahter et al. 2007), though examples are limited for lead specifically. For example, among 195 children in the Cincinnati Lead Study, there was a statistically significant difference by sex in the association between prenatal maternal blood lead concentrations and both attention and visuoconstruction test performance measured at 15–17 years, with significant negative associations seen among boys only (Ris et al. 2004). In experimental studies, immunotoxicity following lead exposure has been shown to be greater in female chickens and rats than in males (Bunn et al. 2000, 2001). An obvious explanation of our sex-specific effects, however, is not clear, and future studies should consider this point specifically.

Walking speed is a marker of general health that may reflect disturbances to the musculoskeletal, nervous, and cardiovascular systems (Abellan van Kan et al. 2009; Atkinson et al. 2007; Baezner et al. 2008; Buchman et al. 2009; Studenski et al. 2011). Clinically, walking speed can be used to predict life expectancy among older adults when coupled with information on age and sex (Hall 2006; Studenski et al. 2011). Currently, there are few studies of effects of environmental toxicants on walking speed. One previous NHANES study of urinary metals and walking speed reported that urinary cobalt, but not urinary lead, was associated with time to walk 20 feet (Lang et al. 2009). However, lead measurements in spot urine samples are a less reliable biomarker of low-level lead exposure than blood lead because of marked within-individual variability (Agency for Toxic Substances and Disease Registry 2007; Navas-Acien et al. 2005). Past epidemiology studies of adults with occupational exposure to lead have reported that blood lead concentrations are associated with detriments in motor performance and with muscle weakness (Baker et al. 1979; Bleecker et al. 2007; Weisskopf et al. 2004). There is also evidence that lead exposure at lower levels affects motor function, although the literature is more limited (Bandeen-Roche et al. 2009; Grashow et al. 2013; Weisskopf et al. 2007).

Lead may influence walking speed through several potential mechanisms. First, walking is often viewed as a rhythmic, simple motor task, but among older adults, it has been shown to draw upon higher-level resources, including executive function (Hausdorff et al. 2005). As a complex motor task, walking may be regulated by neural networks that draw on cognitive resources (Atkinson et al. 2007; Cocchini et al. 2004; Fitzpatrick et al. 2007; Inzitari et al. 2007; Rosano et al. 2005; Sheridan et al. 2003; Soumare et al. 2009b). Lead has been found to be associated with worse cognitive function in adults, which may explain part of the association with walking speed (Bandeen-Roche et al. 2009; Shih et al. 2006, 2007; Weisskopf et al. 2007; Weuve et al. 2009). However, our results did not change meaningfully when we adjusted for the DSST score.

Second, lead has been associated with Parkinson’s disease (Coon et al. 2006; Weisskopf et al. 2010) and motor slowing is one of the hallmarks of the disease; it is possible that in some persons lead exposure may induce subtle parkinsonian signs without reaching clinical criteria for Parkinson’s disease. Parkinson’s disease is rare and even less common in women than in men, and it is therefore unlikely that it accounts entirely for the association between lead and walking speed.

Third, the cerebellum, a brain region that is involved in motor control and coordination, can be a target of lead toxicity. People with a smaller left cerebellum have been reported to walk slower (Rosano et al. 2007). Purkinje cells are the output neurons of the cerebellum, and lead has been shown to induce changes to Purkinje morphology in animal studies (McConnell and Berry 1979; Patrick and Anderson 2000). Atrophy of the cerebellum is associated with gait ataxia, stiff gait, and impaired balance and leg coordination (Morton and Bastian 2003); thus, toxicity of lead to the cerebellum may ultimately affect walking speed. Furthermore, electromyographic studies in workers have shown occupational lead exposure is associated with motor latencies and nerve conduction velocities (Yeh et al. 1995).

Last, another potential pathway is by way of vascular risk factors. Lead has been found to be associated with kidney changes resulting in imbalance of the renin–angiotensin system (Tsaih et al. 2004), and also to alter calcium metabolism resulting in blood pressure elevation, a precursor to hypertension and cardiovascular diseases (Nash et al. 2003; Navas-Acien et al. 2007). These vascular health factors may manifest in slower walking speed (Dumurgier et al. 2009, 2010; Elbaz et al. 2005; Soumare et al. 2009b). In particular, high blood pressure can cause changes to white matter and neuronal circuitry interconnecting the frontal and subcortical areas, regions of the brain associated with decision making and mood. Executive function, depressive symptoms, and walking speed have been shown to be associated with high blood pressure and other vascular risk factors in cohort of elderly community-dwelling people in the Boston, Massachusetts, area (Hajjar et al. 2009). Additionally, peripheral artery disease may also modify walking speed. In our analyses, arthritis, diabetes, heart conditions, and serum homocysteine and C-reactive protein concentrations were significant predictors of walking speed. Adjustment for these comorbid conditions and biomarkers did not substantially alter the association with blood lead (model 3) in women, suggesting that the association is not explained solely through effects of lead on these factors.

Strengths of our study include the large sample that is representative of the U.S. general population ≥ 50 years of age, and information on a multiple potential confounders. There are several limitations to our study as well. First, NHANES has a cross-sectional design, so we cannot make inferences about the temporality of exposure and outcome; in addition, the study does not have data on bone mineral density. Blood lead concentrations can increase as bone mineral density decreases because lead stored in bone is released back into circulation when bone is resorbed (Hu et al. 1998); this could create an association between higher blood lead and slower walking speed if bone loss is associated with slower walking speed. However, there is conflicting evidence for an association between changes in bone mineral density and walking speed, with several studies reporting reduced walking speed with greater bone loss and others no association (Boyer et al. 2011; Kwon et al. 2007; Lindsey et al. 2005; Palombaro et al. 2009; Taaffe et al. 2003). Second, lead was measured in the blood, which has a half-life of roughly 1 month. We do not have information on long-term exposure to lead, such as bone lead measurements (Hu et al. 1998). Finally, there is a potential for selection bias. Nonparticipation in studies has been found to be higher among those who are less healthy and with worse cognitive function and lower socioeconomic status (Goldberg et al. 2001; Launer et al. 1994; Strandhagen et al. 2010). However, because worse health and cognitive function has been associated with slower walking speed (Dumurgier et al. 2009; Hausdorff et al. 2005; Rosano et al. 2005; Sheridan et al. 2003; Studenski et al. 2011), and lower socioeconomic status is usually associated with higher exposure to toxicants such as lead (Evans and Kantrowitz 2002), such selection bias would likely be in the opposite direction of our findings among women, but could contribute to the null findings among men.

Walking speed is increasingly used as a simple and reliable measure of overall motor function in the elderly, and has been shown to be associated with many adverse health events including death (Dumurgier et al. 2009). Our finding of an association between higher blood lead concentration and decreased walking speed in women suggests that lead toxicity may contribute to neuromotor system deficits among the elderly.
